# Making Europe health literate: including older adults in sparsely populated Arctic areas

**DOI:** 10.1186/s12889-022-12935-1

**Published:** 2022-03-16

**Authors:** Sonja S. Gustafsdottir, Arun K. Sigurdardottir, Lena Mårtensson, Solveig A. Arnadottir

**Affiliations:** 1 School of Health Sciences, University of Akureyri, Solborg v/Nordurslod, 600 Akureyri, Iceland; 2 Akureyri Hospital, Akureyri, Iceland; 3 Health and Rehabilitation at the Institute of Neuroscience and Physiology, University of Gothenburg, Gothenburg, Sweden; 4 Department of Physical Therapy, Faculty of Medicine, University of Iceland, Reykjavík, Iceland

**Keywords:** Ageing, Arctic region, Environment, Health literacy, Residence characteristics

## Abstract

**Background:**

Older people have been identified as having lower health literacy (HL) than the general population average. Living in sparsely populated Arctic regions involves unique health challenges that may influence HL. The research aim was to explore the level of HL, its problematic dimensions, and its association with the selection of contextual factors among older adults living in sparsely populated areas in Northern Iceland.

**Method:**

This was a cross-sectional study based on a stratified random sample from the national register of one urban town and two rural areas. The study included 175 participants (57.9% participation rate) who were community-dwelling (40% rural) and aged 65–92 years (*M* 74.2 ± *SD* 6.3), 43% of whom were women. Data were collected in 2017-2018 via face-to-face interviews, which included the standardised European Health Literacy Survey Questionnaire-short version (HLS-EU-Q16) with a score range from 0 to 16 (low-high HL).

**Results:**

The level of HL ranged from 6–16 (*M* 13.25, *SD* ± 2.41) with 65% having sufficient HL (score 13–16), 31.3% problematic HL (score 9–12) and 3.7% inadequate HL (score 0–8). Most problematic dimension of HL was within the domains of disease prevention and health promotion related to information in the media. Univariate linear regression revealed that better HL was associated with more education (*p*=0.001), more resiliency (*p*=0.001), driving a car (*p*=0.006), good access to health care- (*p*=0.005) and medical service (*p*=0.027), younger age (*p*=0.005), adequate income (*p*=0.044) and less depression *(p*=0.006). Multivariable analysis showed that more education (*p*=0.014) and driving a car (*p*=0.017) were independent predictors of better HL.

**Conclusion:**

Difficulties in HL concern information in the media. HL was strongly associated with education and driving a car however, not with urban-rural residency. Mobility and access should be considered for improving HL of older people.

## Background

Health literacy (HL) has been recognised as a critical determinant and moderator of health [1]. It has a broad and inclusive definition, referring to personal skills and social resources needed for individuals and communities to achieve, understand, appraise and use information and services to make well-founded decisions about health [2]. Limited HL is considered to reinforce existing inequalities in health [1].

Older people are of specific concern when it comes to HL [1], as this group has been prominently connected to limited HL [3, 4], which is in turn linked to an increased use of health services and higher mortality rates [5, 6]. Better HL, conversely, is considered a predictive factor for older people’s use of preventive health care [7].

To investigate HL among the older population, the conceptual model behind the International Classification of Functioning, Disability and Health (ICF) can be used [8]. Accordingly, the main aspects of HL are reflected in an individual’s functioning, which indicates the positive attributes of interaction between an individual’s health condition and contextual (environmental and personal) factors. Although ‘health condition’ is commonly used as a term for diseases or disorders, it also includes other circumstances, such as ageing [9]. Therefore, HL among older adults can be viewed as resulting from dynamic interactions between the process of ageing (health condition) and a collection of contextual factors that ‘represent the complete background of people’s life and living’ ([10] p16). Environmental (e.g. various settings, services, support, attitudes and policies) and personal (e.g. gender, age in years, education and other health conditions) contextual factors can act and interact as barriers to or facilitators of HL in old age.

Personal contextual factors that have been prominently linked with low HL are lower education and income [3, 4]. Other personal factors linked with low HL are, for example, lower self-rated health [11] and health conditions like depression [12].

Environmental contextual factors may also play an important role in facilitating or hindering HL in old age. Such factors include where one lives and the surrounding social structure. In the European Union, the proportion of older people living in rural areas is typically higher compared to urban areas [13]. Studies have demonstrated health inequities between urban and rural populations [14, 15], and living in rural areas has been associated with factors such as poor health and disability, less education and lower income [13, 14]. The results from a systematic review indicated that urban populations have better HL than those living in rural areas [15]. In Arctic regions, the demanding environment can also act as a barrier to ideal neighbourhood characteristics for active ageing [16] and ageing in place [17], such as outdoor spaces and buildings, transportation, and social and civic participation. However, some potential facilitators of HL should not be overlooked, as research findings have indicated that the level of resilience in older people in rural settings is high [18].

Another environmental factor that should be considered is ageism in the form of systematic stereotyping of older people and viewing them as a homogeneous group [19] by creating literacy-related barriers to information, services and care [8]. Old-age exclusion has been declared a remaining fundamental challenge for ageing communities in Europe with, for example, exclusion from health services [19]. Programmes aiming to promote better health among older people have been criticised for focusing too much on individual behaviours and ignoring environmental factors like social structures, services, societal attitudes and ideologies [20].

**Fig. 1.**
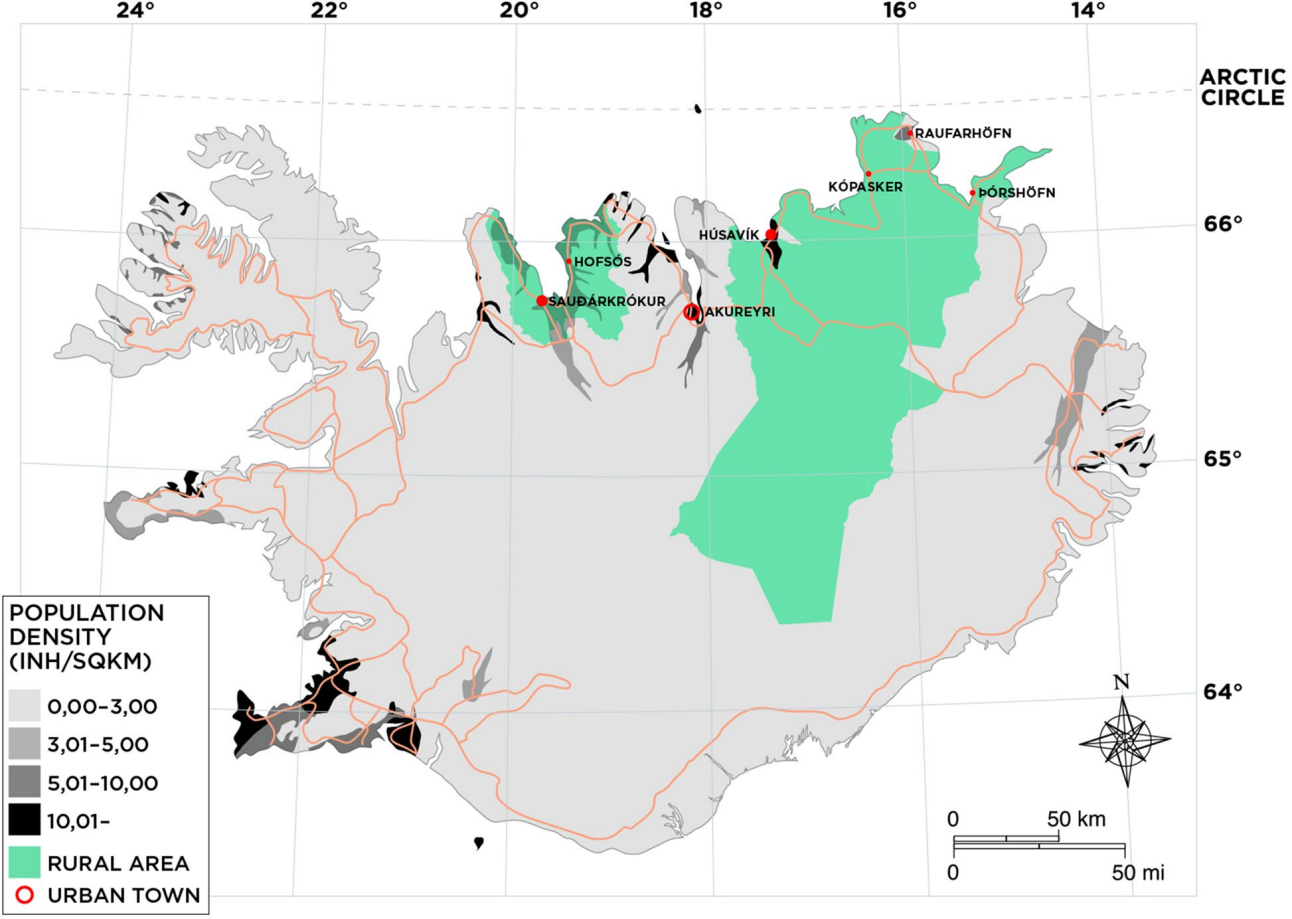
Research areas and population density of Iceland. The green areas represent rural areas (by zip code). The urban town of Akureyri is marked with the largest red circle. The next-largest red circles represent towns (Saudarkrokur, Husavik) in both rural areas of the study that were excluded. The small red circles mark the locations of health clinics in rural areas. Areas shaded in grey and black indicate the population density, with black indicating the most densely populated areas

According to population projections, it is expected that by 2037, 20% of all Icelanders will be 65 years of age or older compared to 14% in 2020, and by 2064, over 25%, with the greatest increase in the oldest group [21]. Moreover, a greater proportion of residents are 65 years or older in rural areas compared to urban areas in Iceland [21]. With about 370,000 inhabitants in an area of 103.000 km^2^, Iceland, which is near the Arctic Circle, is one of the most sparsely populated countries in Europe [22]. The capital area in the south of the country is home to about two-thirds of the population, whereas the remaining third live mostly along the coastline (see Fig. 1). Importantly, in recent years, Icelanders have received more limited health services due to both depopulation in rural municipalities and changes to the public healthcare system following the economic crisis in 2008, which was reorganised to create fewer – yet stronger – healthcare regions [23]. Previous studies have shown that residents in rural areas in Iceland have less education, are more likely to cohabitate, perceive their income as inadequate, and have worse self-rated health and more depressive symptoms compared to urban residents [24–27]; however, they rated their access to medical care as good [28].

There is limited information on levels of HL among older people in sparsely populated areas in Europe and the factors that influence these levels. Therefore, the aim of this study was to (a) collect information about HL levels among an understudied group of older people living in Iceland, a relatively sparsely populated Arctic European region, (b) analyse problematic dimensions of HL, and (c) explore associations between HL levels and contextual factors, including age, gender, education, income, self-evaluated health condition, resilience, urban/rural residency, perceived access and distance to different services, means of transportation, and social participation.

## Methods

This study was part of a larger, population-based research project, which has previously been described in detail [29]. In brief, a stratified random sample from the national register that considered place of residency (by zip codes), age and gender was used in three areas in Northern Iceland. Data were collected from September 2017 until February 2018 via face-to-face interviews. A research group, including multi-disciplinary faculty from two universities in Iceland, conducted the study. The data collection tools included an international standardised instrument for HL, three international standardised instruments for selected contextual factors, and a collection of single-item questions for additional contextual factors.

### Participants and the research area

The study included people 65 years of age and older living in their own homes in three distinct geographical areas in Northern Iceland closest to the Arctic Circle (see Fig. 1). One urban town and two nearest rural districts were included: (a) Akureyri, the largest urban town outside of the capital area and, with 19,000 inhabitants, a university, a secondary national hospital and diverse services; (b) Skagafjordur district, with around 4000 inhabitants, a primary healthcare centre and a small hospital in the town of Saudarkrokur (not included in the study); and (c) Thingeyjarsyslur district, with around 4000 inhabitants, a primary healthcare centre and a small hospital in the town of Husavik (not included in the study). These three areas were selected as they represent parts of Iceland with understudied older populations and because they fulfilled predetermined definitions for urban/rural residency in Iceland [30]. Power analyses based on a study by Arnadottir [24] showed that at least 250 participants are needed to obtain a statistically valid difference between urban/rural participants. Therefore, a stratified random sample from the national registry (*N*=400) based on community size was taken, with 240 from the urban town and 160 from the two rural areas, with equal gender ratio. The rural group was oversampled in order to optimize reliable estimates for that part of the total sample and to have the two sub-samples as similar in size as possible. Moreover, to be included, residents had to be able to verbally communicate in Icelandic and to determine a time for a face-to-face interview. Twenty residents did not meet the inclusion criteria, five had passed away and 73 could not be reached, resulting in a sample of 302 potential participants.

### Participant selection

An introduction letter, with information about the study, was sent to all potential participants. Two weeks later, they received a telephone call from a research assistant (four third-year nursing students specifically trained for data collection) asking whether they would be willing to participate in the study. If they decided to participate, they were asked to determine a time and date for a face-to-face interview. The participants could choose to meet with the research assistant at their own home, at the research centre (based in Akureyri) or at the nearest healthcare centre. Of the 302 potential participants, 175 (57.9%) agreed to participate. Those who declined to participate did not differ significantly from the study sample in terms of age (*p*=0.77) or residency (*p*=0.55) – more women or 73, however, declined to participate compared to 44 men (*p*=0.01). Those who declined to participate were asked to provide an explanation for their decision. The most common explanation was that they were too busy to participate or had recently taken part in other studies.

### Assessment of health literacy and contextual factors

To measure HL, the 16-item validated Icelandic version of the European Health Literacy Survey Questionnaire-short version (HLS-EU-Q16-IS) which presents sound psychometric properties, with Cronbach´s alpha 0.88 [31] was used. The instrument was developed by a consortium of eight European countries [32] and is based on a conceptual model of HL outlining its main dimensions. The core of the model conceives the key processes of accessing, understanding, appraising and applying health-related information within three domains: health care, disease prevention and health promotion. The domain of health care refers to medical or clinical issues; the domain of disease prevention includes information on risk factors for poor health; and the domain of health promotion includes determinants of health in the social and physical environment. Each of the items on the HLS-EU-Q16-IS is rated on a four-point scale: ‘very difficult’, ‘fairly difficult’, ‘fairly easy’ and ‘very easy’. Each response is then dichotomised into ‘easy’ (scored with 1) and ‘difficult’ (scored with 0) [33]. Summing these responses gives the final score of the HLS-EU-Q16, which can range from 0 (low/no HL) to 16 (high HL). Missing responses, on one or two items, are replaced with 0 before calculating the total. If there are more than two missing responses, the total score cannot be calculated. The HLS-EU-Q16 was selected as it is one of the few HL instruments designed to measure HL levels among the general population rather than specific patient groups, is internationally recognised [4, 32] and available in the Icelandic language.

Resilience was measured with the Connor-Davidson Resilience Scale (CD-RISC), which contains 25 items scored from 0 to 4 [34]. The scoring of the scale is based on summing the total of all items. Final scores range from 0 to 100, with higher scores reflecting greater resilience. The original version of the scale has presented sound psychometric properties [34] also in the settings of measuring resilience in older adults [35] the psychometric properties of the Icelandic translation have not been published.

Depression was measured with the Geriatric Depression Scale (GDS), which contains 30 items scored from 0 to 1 [36]. The scoring of the scale is based on summing the total of all items. Final scores range from 0 to 30, with higher scores indicating more severe depression. The original version of GDS [37] and the Icelandic translation [38] have demonstrated sound psychometric properties within older community-dwelling populations

Self-rated health (SRH) was measured using a standardised single-item question on global health status: ‘How would you rate your overall health?’ [39]. Response options were as follows: 5 – very good, 4 – good, 3 – fair, 2 – bad and 1 – very bad. The scores range from 1 to 5, with a higher score reflecting higher self-rated health. The measurement is considered valid and reliable at the population level among people without cognitive impairment, is critically used internationally [40, 41] and in Iceland [29].

In addition to measures based on standardised instruments, participants answered singe-item questions on personal and environmental contextual factors. Personal factors were age in years, age group (65 to 74 and 75 to 92 years old), gender, educational level and years in school, monthly income, and whether income was adequate. Environmental factors were urban/rural residency, distance and perceived access to healthcare services, recreational centres and organised physical training, perceived access to medical services, distance to a store, means of transportation, and social connections in the form of cohabitating or living alone, having someone to ask for assistance, frequency of meeting children or other relatives, and frequency of meeting friends or neighbours.

### Statistical analysis

Descriptive statistics for the contextual factors, describing the background of all study participants, included mean (*M*) and standard deviation (*SD*) for continuous variables and counts and proportions for categorical variables. Descriptive statistics were also used to present HL levels. To interpret the final score on the HL scale, three HL levels have been defined: inadequate HL (0 to 8), problematic HL (9 to 12) and sufficient HL (13 to 16) [33]. To compare personal and environmental factors by residency, independent t-tests were used for continuous variables and chi-square tests were used for categorical variables. A few univariate linear regression models were used to analyse the association between HL (dependent variable) and contextual factors (independent variables). In addition, one multivariable linear regression model was used to describe the association between HL (dependent variable) and a selection of both personal and environmental contextual factors (independent variable) with significant (*p*-value <0.05) bivariate associations from the univariate linear regression. For statistical purposes and to avoid collinearity, the independent variables age in years and education in years (interval-ratio scale) were used in the multivariable mode rather than age groups and education level (ordinal scale). Statistical analysis was run with the IBM SPSS software package, v22 [42].

### Ethical approval

The study was approved by the Icelandic National Bioethics Committee (VSNb2016060007/03.01) and reported to the Icelandic Data Protection Authority. Written informed consent was obtained from all participants.

## Results

### Participants

Participants’ contextual factors, personal and environmental, are shown in Table 1. Compared to participants in rural areas, residents in the urban town had higher education levels, showed more resilience, used public transportation more often, met friends and neighbours daily more often and rated access to recreational centres and organised physical training better. Interestingly, however, they rated access to medical services worse than those living in rural areas.

**Table 1:** Participants’ contextual factors according to residency

Personal factors		Total ***N***=175	Rural ***n***=70	Urban ***n***=105	***p***-value*
Age in years, mean (*SD*), [min-max]		74.2 (6.3) [66–92]	73.9 (6.2) [66–89]	74.4 (6.4) [66–92]	0.550
Age groups, *n* (%)	65–74 years	104 (59.4)	43 (61.4)	61 (58.1)	0.660
	75–92 years	71 (40.6)	27 (38.6)	44 (41.9)	
Gender, *n* (%)	Female	75 (43)	33 (47)	42 (40)	0.350
	Male	100 (57)	37 (53)	63 (60)	
Years in school, mean (*SD*), [min-max]		11.1 (5.3) [0–30]	9.0 (4.7) [0–24]	12.5 (5.2) [1–30]	<0.001**
Education level, *n* (%)	Elementary	78 (45.1)	38 (55.9)	40 (38.1)	0.044**
	Secondary/trade school	66 (38.2)	23 (33.8)	43 (41)	
	University degree	29 (16.8)	7 (10.3)	22 (21)	
Income per month, *n* (%)	<1.440 EUR	8 (5)	4 (6.9)	4 (4)	0.627
	1.440–2.885 EUR	74 (46.5)	28 (48.3)	46 (45.5)	
	>2.885 EUR	77 (48.4)	26 (44.8)	51 (50.5)	
Adequate income, *n* (%)	Yes	132 (75.4)	53 (75.7)	79 (75.2)	0.943
	No	43 (24.6)	17 (24.3)	26 (24.8)	
Working or retired, *n* (%)	Working	57 (32.6)	36 (51.4)	21 (20)	<0.001**
	Retired	118 (67.4)	34 (48.6)	84 (80)	
HLS-EU-Q16, mean (*SD*), [min-max]		13.2 (2.4) [6–16]	13.0 (2.4) [6–16]	13.4 (2.4) [7–16]	0.464
GDS, mean (*SD*), [min-max]		4.9 (3.8) [0–20]	4.9 (4.1) [0–20]	4.9 (3.7) [0–18]	0.978
CD-RISC, mean (*SD*), [min-max]		75.9 (12.4) [39–100]	70.4 (10.9) [39–94]	79.3 (12.0) [50–100]	<0.001**
SRH, mean (*SD*), [min-max]		3.0 (0.9) [1–5]	3.0 (0.8) [2–5]	3.0 (0.9) [1–5]	0.833
**Environmental factors**		**Total** ***N*=175**	**Rural** ***n*=70**	**Urban** ***n*=105**	*p*-value*
Way of living, *n* (%)	Cohabitating	135 (77.1)	59 (84.3)	76 (72.4)	0.066
	Living alone	40 (22.9)	11 (15.7)	29 (27.6)	
Means of transportation, *n* (%)	Walk	118 (67.4)	47 (67.1)	71 (67.6)	0.947
	Drive on own	162 (92.6)	64 (91.4)	98 (93.3)	0.638
	Driven by others	39 (22.3)	13 (18.5)	26 (24.7)	0.749
	Public transport	21 (12)	3 (4.3)	18 (17.1)	0.010**
Have someone to ask for assistance, *n* (%)	Yes	170 (97.1)	69 (98.6)	101 (96.2)	***
	No	5 (2.9)	1 (1.4)	4 (3.8)	
How often meet children or other relatives, *n* (%)	Daily	69 (39.7)	30 (43.5)	39 (37.1)	0.481
	Weekly	70 (40.2)	19 (27.5)	51 (48.6)	
	Monthly	26 (14.9)	16 (23.2)	10 (9.5)	
	Yearly	9 (5.2)	4 (5.8)	5 (4.8)	
How often meet friends or neighbours, *n* (%)	Daily	60 (34.3)	16 (22.9)	44 (41.9)	0.003**
	Weekly	97 (55.4)	43 (61.4)	54 (51.4)	
	Monthly	15 (8.6)	9 (12.9)	6 (5,7)	
	Yearly	3 (1.7)	2 (2.9)	1 (1)	
Perceived access to healthcare services, *n* (%)	Good	99 (56.6)	38 (54.3)	61 (58.1)	0.156
	Rather good	47 (26.9)	26 (37.1)	21 (20)	
	Neither nor	7 (4)	2 (2.9)	5 (4.8)	
	Rather bad	16 (9.1)	3 (4.3)	13 (12.4)	
	Bad	6 (3.4)	1 (1.4)	5 (4.8)	
Perceived access to medical services, *n* (%)	Good	104 (59.4)	41 (58.6)	63 (60)	0.022**
	Rather good	41 (23.4)	22 (31.4)	19 (18.1)	
	Neither nor	8 (4.6)	3 (4.3)	5 (4.8)	
	Rather bad	16 (9.1)	2 (2.9)	14 (13.3)	
	Bad	6 (3.4)	2 (2.9)	4 (3.8)	
Perceived access to recreational centres, *n* (%)	Good	89 (50.9)	23 (32.9)	66 (62.9)	<0.001**
	Rather good	23 (13.1)	11 (15.7)	12 (11.4)	
	Neither nor	56 (32)	31 (44.3)	25 (23.8)	
	Rather bad	6 (3.4)	4 (5.7)	2 (1.9%)	
	Bad	1 (0.6)	1 (1.4)	0 (0)	
Perceived access to organised physical training, *n* (%)	Good	69 (39.4)	16 (22.9)	53 (50.5)	<0.001**
	Rather good	24 (13.7)	10 (14.3)	14 (13.3)	
	Neither nor	69 (39.4)	32 (45.7)	37 (35.2)	
	Rather bad	9 (5.1)	8 (11.4)	1 (1)	
	Bad	4 (2.3)	4 (5.7)	0 (0)	
Distance from healthcare services, *n* (%)	0–5 km	107 (61.1)	4 (5.7)	103 (98.1)	<0.001**
	5–20 km	21 (12)	19 (27.1)	2 (1.9)	
	20 km+	47 (26.9)	47 (67.1)	0 (0)	
Distance from recreational centres, *n* (%)	0–5 km	123 (72.4)	18 (27.7)	105 (100)	<0.001**
	5–20 km	32 (18.8)	32 (49.2)	0 (0)	
	20 km+	15 (8.8)	15 (23.1)	0 (0)	
Distance from a store, *n* (%)	0–5 km	119 (68)	14 (20)	105 (100)	<0.001**
	5–20 km	29 (16.6)	29 (41.4)	0 (0)	
	20 km+	27 (15.4)	27 (38.6)	0 (0)	
Distance from organised physical training, *n* (%)	0–5 km	124 (73.8)	19 (30.2)	105 (100)	<0.001**
	5–20 km	20 (11.9)	20 (31.7)	0 (0)	
	20 km+	24 (14.3)	24 (38.1)	0 (0)	

*HLS-EU-Q16 *European Health Literacy Survey Questionnaire-short version, *GDS *Geriatric Depression Scale, *CR-RISC *Connor-Davidson Resilience Scale, *SRH *Self-rated health-single item question. *Differences between rural/urban residents were based on *t*-test for continuous variables and a chi-square test for categorical variables. **Statistical difference, *p*<0.05. ***Differences between rural/urban residents could not be calculated due to the homogeneity of responses

### Health literacy

Of the total 175 participants, 134 completed the questionnaire with less than two missing answers. Scores ranged from 6 to 16, with a mean of 13.25 (*SD* ± 2.41) and a median of 13. Eighty-seven (65%) scored from 13 to 16, indicating sufficient HL, 42 (31.3%) scored from 9 to 12, which has been defined as problematic HL, and 5 (3.7%) scored from 0 to 8, indicating inadequate HL. See Table 2 for more information about each item. The items most often rated as difficult by participants were in the domain of disease prevention and health promotion, related to information in the media. Conversely, the items most often rated as easy were predominantly in the domain of health care.

**Table 2:** Distribution of answers to the HLS-EU-Q16 items

Item	Domain*	*n*	Easy *n* (%)	Difficult *n* (%)	Missing *n* (%)
1. find information about treatments for illnesses that concern you?	HC	170	148 (87)	22 (13)	5 (3)
2. find out where to get professional help when you are ill?	HC	174	163 (93)	11 (7)	1 (0.5)
3. understand what your doctor says to you?	HC	175	170 (97)	5 (3)	0 (0)
4. understand your doctor´s or pharmacist’s instructions on how to take a prescribed medicine?	HC	174	171 (98)	3 (2)	1 (0.5)
5. judge when you may need to get a second opinion from another doctor?	HC	157	127 (80)	30 (20)	18 (10)
6. use information the doctor gives you to make decisions about your illness?	HC	168	163 (97)	5 (3)	7 (4)
7. follow instructions from your doctor?	HC	174	169 (97)	5(3)	1 (0,5)
8. find information about how to manage mental health problems like stress or depression?	DP	145	110 (76)	35 (24)	30 (17)
9. understand health warnings about behaviour such as smoking, low physical activity and drinking too much?	DP	174	168 (96)	6 (4)	1 (0.5)
10. understand why you need health screenings?	DP	175	171 (98)	4 (2)	0 (0)
11. judge if the information about health risks in the media is reliable?	DP	171	73 (43)	98 (57)	4 (2)
12. decide how can protect yourself from illness based on information in the media?	DP	168	62 (37)	106 (63)	7 (4)
13. find out about activities that are good for your mental well-being?	HP	172	146 (85)	26 (15)	3 (1,5)
14. understand advice on health from family members or friends?	HP	171	144 (84)	27 (16)	4 (2)
15. understand information in the media about how to get healthier?	HP	172	87 (51)	85 (49)	3 (1,5)
16. judge which everyday behaviour is related to your health?	HP	175	162 (93)	12 (7)	0 (0)

Domain*: *HC *Health care, *DP *Disease prevention, *HP *Health promotion

### Health literacy and contextual factors

The results from the univariate linear regression are shown in Table 3. A negative significant difference in HL was found within five personal factors: age in years *F*(1.132)=8.3; age groups *F*(1.132)=7.6; education level *F*(1.131)=12.5; having adequate income to fulfil needs *F*(1.132)=4.1; depression *F*(1.131)=7.8. A positive significant difference in HL levels was found within two personal factors: education in years *F*(1.112)=11.4; resilience *F*(1.127)=10.9. A positive significant difference in HL levels was found within three environmental factors: transportation by driving a car on one’s own *F*(1.132)=7.9; perceived access to healthcare services *F*(1.132)=8.0; perceived access to medical services *F*(1.132)=4.9.

**Table 3:** Health literacy and its association with personal and environmental contextual factors

Contextual factors	Univariate associations			A multivariable model		
Personal factors	***β*** (***t***-value)	95% CI Lower Upper	***p***-value	***β*** (***t***-value)	95% CI Lower Upper	***p***-value
Age in years	-0.244 (-2.887)	-0.157 -0.029	0.005*	-0.155 (-1.672)	-0.144 0.012	0.098
Age group (75–92 years)	-0.234 (-2.765)	-1.985 -0.329	0.007*			
Gender (male)	-0.103 (-1.191)	-1.327 0.330	0.236			
Years in school	0.304 (3.378)	0.057 0.221	0.001*	0.236 (2.508)	0.022 0.186	0.014*
Education level (secondary/trade school)	0.129 (1.488)	-0.208 1.470	0.139			
Education level (elementary)	-0.295 (-3.540)	-2.254 -0.638	0.001*			
Income per month (<1.440 EUR)	-0.051 (-0.567)	-2.828 1.569	0.572			
Income per month (1.440–2885 EUR)	-0.118 (-1.329)	-1.425 0.280	0.186			
Adequate income (no)	-0.174 (-2.030)	-1.915 -0.025	0.044*	-0.061 (-0.650)	-1.330 0.673	0.517
Working or retired (retired)	-0.112 (-1.297)	-1.498 0.312	0.197			
GDS	-0.237 (-2.794)	-0.248 -0.042	0.006*	-0.113 (-1.125)	-0.187 0.052	0.263
CD-RISC	0.281 (3.305)	0.022 0.087	0.001*	0.119 (1.203)	-0.015 0.061	0.232
SRH	-0.165 (-1.922)	-0.876 0.013	0.057			
**Environmental factors**	***β*** **(*t*-value)**	**95% CI** **Lower** **Upper**	***p*-value**	***β*** **(*t*-value)**	**95% CI** **Lower** **Upper**	***p*-value**
Residency (urban)	0.064 (0.735)	-0.556 1.212	0.464			
Way of living (living alone)	-0.036 (-0.409)	-1.170 0.769	0.683			
Means of transportation:						
Walking	-0.041 (-0.469)	-1.088 0.671	0.640			
Driving a car	0.239 (2.822)	0.685 3.893	0.006*	0.213 (2.424)	0.489 4.906	0.017*
Public transport	0.069 (0.794)	-0.724 1.695	0.429			
Driven by others	-0.054 (-0.619)	-1.464 0.766	0.537			
How often meet children or other relatives	-0.113 (-1.300)	-0.803 0.166	0.196			
How often meet friends or neighbours	0.039 (0.449)	-0.509 0.808	0.654			
Perceived access to healthcare services	0.239 (2.830)	0.155 0.875	0.005*	0.252 (1.734)	-0.076 1.131	0.086
Perceived access to medical services	0.191 (2.231)	0.045 0.748	0.027*	0.036 (0.253)	-0.492 0.636	0.801
Perceived access to recreational centres	0.088 1.020)	-0.204 0.638	0.310			
Perceived access to organised physical training	0.164 1.914)	-0.013 0.761	0.058			
Distance from healthcare services	-0.116 (-1.343)	-0.874 0.167	0.181			
Distance from recreational centres	0.095 (1.086)	-0.338 1.162	0.280			
Distance from a store	0.020 (0.231)	-0.567 0.717	0.818			
Distance from organised physical training	-0.072 (-0.822)	-0.951 0.393	0.413			

*GDS* Geriatric Depression Scale, *CD-RISC *Connor-Davidson Resilience Scale, *SRH *Self-rated health-single item question. *Statistical significance *p*<0.05

In multivariable linear regression, years in school and the ability to drive a car on one’s own were independent predictors of HL. The model was significant *F*(10.100)=5.0, *p*<0.001, *R*^*2*^ 0.289, explaining 29% of the HL variable.

## Discussion

This study contributes to the currently limited literature on the HL of older people in sparsely populated Arctic regions – in this case, one urban town and two rural areas in Iceland. Our results indicated that HL levels were lower among the population in this study compared to the general population of Icelanders [31]. Of the study participants, 35% demonstrated problematic or inadequate HL in contrast to 27.5% of the general public [31]. Therefore, the importance of addressing HL specifically among older people is emphasized. The results revealed that HL decreased with advanced age, supporting the heterogeneity of older people, and that HL becomes more problematic with increased age. The reason for this could be multidimensional and complex, including barriers associated with the process of ageing and a decline in body function. However, ageism in the form of societal attitudes should be considered. For example, increased use of technology and a limited consideration of various environmental factors, like culture, natural environment and services can result in exclusion from information and services.

The findings indicate that the most problematic dimension of HL is within the domains of disease prevention and health promotion rather than that of health care. The role of the general practitioner (GP) and the primary healthcare system seems to be well recognised. People know where to look for assistance in the case of illness or disease however, this is not the case regarding how to maintain or promote one’s own health. In a Danish study, Bo et al. [43] reported fewer difficulties engaging with healthcare providers and understanding health information with increased age. This could be explained with the established relationship between people and their GP, as part of the countries public healthcare system is provision of GP´s to all citizens in their geographical regions. Previous findings from rural Iceland are corresponding [28], which might be related to the fact that Nordic countries have similar healthcare systems. The results of this study also revealed specific difficulties in HL concerning understanding, appraising and using health-related information in the media. Most participants found it difficult to determine how to protect themselves from illnesses based on information in the media. This accords with findings from a study on HL among older people in Finland [12]. At present, in the context of the ongoing COVID-19 pandemic, older adults may experience being excluded from precautionary measures which are extensively presented via technology.

The findings from this study found no difference in HL in terms of urban-rural residency. According to Aljassim and Ostini [15], rurality alone may not be a determinant of HL, as other personal and environmental factors might play important roles in a complex interplay within both HL and rurality. HL, in this study, increases with younger age, more education, having adequate income, less depression, increased resilience, driving a car and good self-reported access to medical and healthcare services. Multivariable analysis revealed that driving a car and having more years of education were independent predictors of better HL. It should not be overlooked, that driving a car may largely influence HL, as it is directly linked to physical access to health-related information and services. This ability may afford older people more independence and enhance their inclusion in sparsely populated Arctic regions. Good perceived access to healthcare and medical services were also related to a higher HL score. Access to medical services was perceived to be better in the two rural areas than in the urban town, which aligns with findings from other rural areas in Iceland [28]. Access might, therefore, not only be limited to ground distance but may also refer to social access, costs, waiting time and knowing where to seek or access information. Although sparsely populated rural communities might lack public transport, they may also have less complex communication pathways and more personal connections between healthcare staff and residents than in urban areas [28].

In this study while significant in the univariate analysis, income level was not independently associated with HL in the multivariable analysis like years of education. However, higher education is generally linked to higher income. The importance of education and income to HL is particularly interesting considering that Iceland is a high-income country [44] with comparatively high equity in education [45]. Older people with higher levels of education and income have better HL [1] – both factors, yet, are not represented among those living rurally, internationally [13–15] and in Iceland [24–27] thereby, indicating that there are vulnerable groups within every country, regardless of the economy.

In this study, overall, resilience can be considered relatively high and depression symptoms limited, although HL increased with higher resilience and decreased with more depression symptoms. Resilience was, however, higher in the urban town in this study, compared to previous findings that indicated higher resilience related to older people living rurally [18]. Better HL is considered to increase resilience if combined with, for example, better access to health information and services [46]. The link and interactions between HL, resilience and contextual factors need to be investigated further, specifically in rural areas.

### Strengths and limitations

The strength of this research was its use of an international standardised instrument to measure HL as well as three other international standardised instruments. Yet, it might be considered a limitation that the psychometric properties of the Icelandic translation of the CD-RISC have not been published. The face-to-face interviews used for data collection generally minimised non-responses and maximised the quality of the collected data, for example, the interviewer could clarify items to the respondent if needed. However, having described the HLS-EU-Q16-IS instrument in the methods section and the four- point response scale that does not include options like “I don’t know/I don’t want to answer” and the fact that participants signed an informed consent stating voluntary participation could explain the missing answers for some items. The study sample was relatively small, yet reasonable compared to the size of the general population and the population-level random sampling approach used. For unknown reasons, women were more likely to decline participation than men and were therefore slightly underrepresented compared to the general population.

## Conclusion

In a dynamic and complex interaction, HL among older adults is a result of the process of aging and contextual factors that act and interact as barriers or facilitators. Although urban/rural residency does not seem to influence HL, other factors do, like age, education and income, depression, resilience, driving a car and access to medical and healthcare services, with more education and driving a car being the strongest associated factors. Thus, a one-size-fits-all approach does not apply to measures taken to increase HL, and we therefore need to consider and respond to the ways in which older people are being excluded from information and services by, for example, increased use of technology and the need for better strategies to improve the health literacy of those who may be less mobile. Particular attention should be paid to disease prevention and health promotion and to the role of the media in providing reliable health-related information. In a call for action within the European Union to improve HL, identifying specific barriers among population groups that need more support was highlighted. This study did so by providing information on HL levels among an understudied group of older people in sparsely populated Arctic regions and demonstrating the importance of associations with various contextual factors.

## Data Availability

The datasets used and/or analysed during the current study and the Icelandic version of the questionnaire are available from the corresponding author on reasonable request.

## Abbreviations

HL: Health Literacy
HLS-EU-Q16: The European Health Literacy Survey Questionnaire- short version
HLS-EU-Q16-IS: Icelandic version of the European Health Literacy Survey Questionnaire-short version
CD-RISC: Connor-Davidson Resilience Scale
GDS: Geriatric Depression Scale
SRH: Self-rated health-single item question
HC: Health care
DP: Disease prevention
HP: Health promotion
GP: General practitioner
